# NLRP3 Inflammasome Activation in Dialyzed Chronic Kidney Disease Patients

**DOI:** 10.1371/journal.pone.0122272

**Published:** 2015-03-23

**Authors:** Simona Granata, Valentina Masola, Elisa Zoratti, Maria Teresa Scupoli, Anna Baruzzi, Michele Messa, Fabio Sallustio, Loreto Gesualdo, Antonio Lupo, Gianluigi Zaza

**Affiliations:** 1 Renal Unit, Department of Medicine, University-Hospital of Verona, Verona, Italy; 2 Interdepartmental Laboratory for Medical Research (LURM), University of Verona, Verona, Italy; 3 Department of Pathology and Diagnostics, Section of General Pathology, University of Verona, Verona, Italy; 4 Department of Emergency and Organ Transplantation-Nephrology, Dialysis and Transplantation Unit, University of Bari, Bari, Italy; University of California Merced, UNITED STATES

## Abstract

To assess whether NLR pyrin domain-containing protein 3 (NLRP3) inflammasome, a multiprotein complex that mediates the activation of caspase-1 (CASP-1) and pro-inflammatory cytokines IL-18 and IL-1β, could be involved in the chronic inflammatory state observed in chronic kidney disease patients undergoing hemodialysis treatment (CKD-HD), we employed several biomolecular techniques including RT-PCR, western blot, FACS analysis, confocal microscopy and microarray. Interestingly, peripheral blood mononuclear cells from 15 CKD-HD patients showed higher mRNA levels of *NLRP3*, *CASP-1*, *ASC*, *IL-1β*, *IL-18* and *P2X7receptor* compared to 15 healthy subjects. Western blotting analysis confirmed the above results. In particular, active forms of CASP-1, IL1-β and IL-18 resulted significantly up-regulated in CKD-HD *versus* controls. Additionally, elevated mitochondrial ROS level, colocalization of NLRP3/ASC/mitochondria in peripheral blood mononuclear cells from CKD-HD patients and down-regulation of CASP-1, IL1-β and IL-18 protein levels in immune-cells of CKD-HD patients stimulated with LPS/ATP in presence of mitoTEMPO, inhibitor of mitochondrial ROS production, suggested a possible role of this organelle in the aforementioned CKD-associated inflammasome activation. Then, microarray analysis confirmed, in an independent microarray study cohort, that *NLRP3* and *CASP-1*, along with other inflammasome-related genes, were up-regulated in 17 CKD-HD patients and they were able to clearly discriminate these patients from 5 healthy subjects. All together these data showed, for the first time, that NLRP3 inflammasome was activated in uremic patients undergoing dialysis treatment and they suggested that this unphysiological condition could be possibly induced by mitochondrial dysfunction.

## Introduction

Chronic kidney disease (CKD) is one of the leading clinical features of nephrology patients and it represents a major and growing challenge for healthcare systems. The prevalence rate of CKD is increasing worldwide [1–3], primarily as a consequence of the augmented incidence of diabetes, hypertension, and aging population [4–6].

During this condition patients experience significant and progressive biological dysfunctions associated with considerable changes in energy metabolism, nitrogen balance, protein-energy malnutrition and insulin resistance and with a significant increase in the generation of mediators of inflammation/oxidative stress [7–9].

An international consensus categorized CKD into five stages according to the glomerular filtration rate (GFR) [10]. In the last stage of renal failure (end stage renal disease) these alterations are incompatible with life and renal replacement therapies (RRT: hemodialysis, peritoneal dialysis and renal transplantation) are required.

Hemodialysis, most employed RRT worldwide, may induce additional biochemical alterations caused by the contact of blood with dialysis devices [11,12]: phagocyte cells activation [13,14], complement activation, cytokine synthesis and release, oxidative stress [15–18].

Oxidative stress and chronic inflammation, being strongly related, contribute to long-term complications in HD patients, such as cardiovascular disease, atherosclerosis, anemia and malnutrition [19–23].

In the last years, different therapeutic interventions have been proposed to prevent or at least control HD-related cellular/biological alterations, including the use of biocompatible membranes able to limit the complement activation and cytokines release; the activation of high flux dialysis or hemofiltration that would reduce the circulating levels of complement activating molecules and the accurate use of sterile water [24,25]. Moreover, the dialysis devices and membranes (dialysis filters) have continuously modified to improve the biocompatibility profile of the material. However, at the moment, we are still far from the development of full biocompatible dialysis procedure.

Therefore, the comprehension of the mechanisms underlying CKD/HD-related oxidative stress and chronic inflammation may help to achieve this objective. In this context mitochondria being involved in immune response as well as in oxidative stress could have a role.

These organelles are the “powerhouse of the cell” involved in a lot of signaling pathways: oxidative phosphorylation system for the production of the vast majority of intracellular ATP; calcium homeostasis [26]; heme and steroid biosynthesis [27,28] and apoptosis [29].

In addition, as recently published, mitochondria orchestrate innate immune response [30,31] that constitutes the first line of host defense against infection or noxious insults. In order to recognize and respond to microbial substances or molecules that arise during tissue damage or cellular stress, the innate immune system possesses a large number of soluble (e.g. pentraxins), membrane-bound (e.g. Toll-like receptors) and cytosolic (e.g. Nod-like receptors) receptors known collectively as pattern recognition receptors [32].

The Nod-like receptors or nucleotide-binding domain leucine-rich repeat-containing receptors (NLRs), are a family of intracellular immune receptors with more than 20 members known in humans [33,34]. The NLR pyrin domain-containing proteins (NLRP) constitutes an homogenous NLR subfamily [35]. Among these the most studied is NLRP3, that interacts with apoptosis-associated speck-like protein containing a CARD domain (ASC) and with pro-caspase-1 forming the inflammasome [24,31]. The inflammasome mediates the activation of caspase-1, which promotes secretion of the pro-inflammatory cytokines interleukin 1β (IL-1β) and IL-18 [36,37].

A wide array of signals (viruses, bacteria, molecules released during or indicative of tissue injury, environmental origin irritants) [38–40], are able to activate NLRP3 inflammasome but the exact mechanism is poorly understood. Currently, it is well accepted that two steps are required. The first, or priming signal, converges on the activation of NFkB and transcriptional induction of inflammasome components including NLRP3 itself and pro-IL-1β. The second, or activating signal, is able to directly activate inflammasome assembly [41].

Although several reports have focused on inflammasome in various kidney diseases [42,43] no report has studied its role in chronic inflammation associated with hemodialysis. Therefore the aim of our study was to investigate the activation of NLRP3 inflammasome in peripheral blood mononuclear cells (PBMCs) from CKD-HD patients and the possible involvement of mitochondrial dysfunction in this process.

## Methods

### Patients and Controls

A total of 52 subjects [n: 32 hemodialyzed chronic kidney disease patients (CKD-HD) and n:20 healthy subjects (NORM)], after signing informed consent, were enrolled in our study.

Thirty subjects were included in the training-group (15 NORM and 15 CKD-HD) and 22 in the microarray-group (5 NORM and 17 CKD-HD).

Since we did not obtain sufficient number of cells for all the analysis and in order to avoid additional blood collection, western blot experiments and FACS analysis were performed in 10 NORM and 10 CKD-HD.

The main demographic and clinical features are summarized in Table 1.

**Table 1:** Demographics and clinical characteristics of study groups.

	Training-group			Microarray-group		
	NORM	CKD-HD	*P-value*	NORM	CKD-HD	*P-value*
**Number**	**15**	**15**	**/**	**5**	**17**	**/**
**Gender (Male/Female)**	**4/11**	**7/8**	***n*.*s*.**	**2/3**	**10/7**	***n*.*s*.**
**Age (years)**	**48±11.27**	**50.61±7.24**	***n*.*s*.**	**50.78±10.45**	**52.86±8.63**	***n*.*s*.**
**Cause of CKD: GN/ADPKD/RVD/unknown**	**/**	**7,2,2,4**	***n*.*s*.**	**/**	**7,4,1,5**	***n*.*s*.**
**Time on dialysis (years)**	**/**	**4.01±0.42**	**n.s.**	**/**	**3.98±0.56**	**n.s.**
**BMI (kg/m2)**	**22.8±0.64**	**21.7±0.96**	***p<0*.*01***	**23.1±0.82**	**22.1±1.01**	***p<0*.*01***
**Systolic blood pressure (mmHg)**	**121±3.71**	**138±8.11**	***p<0*.*01***	**120±4.01**	**138±8.11**	***p<0*.*01***
**Diastolic blood pressure (mmHg)**	**75.28±4.26**	**89±10.93**	***p<0*.*01***	**74.36±5.31**	**88±10.86**	***p<0*.*01***
**Total protein (g/dl)**	**6.87±0.49**	**5.98±0.34**	***p<0*.*01***	**6.98±0.41**	**6.02±0.31**	***p<0*.*01***
**Albumin (g/dl)**	**4.1±1.56**	**3.74±0.48**	***p<0*.*01***	**4.3±0.31**	**3.48±0.51**	***p<0*.*01***
**Hs-CRP (ng/ml)**	**0.66±0.3**	**12.48±6.4**	***p<0*.*01***	**0.56±0.3**	**11.21±4.2**	***p<0*.*01***
**Hemoglobin (g/dl)**	**13.12±1.49**	**11.12±2.28**	***p<0*.*01***	**13.46±1.62**	**11.48±1.98**	***p<0*.*01***

GN: Glomerulonephritis; ADPKD: Autosomal dominant polycystic kidney disease; RVD: Renal vascular diseases; BMI: Body mass index; CRP: C reactive protein. Values are expressed as mean±SD. P-values calculated by T-Test.

To avoid confounding factors, all patients suffering from systemic autoimmune disorders, (e.g. SLE, vasculitis) infectious diseases, diabetes, chronic lung diseases, neoplasm, or inflammatory diseases and patients receiving antibiotics, corticosteroids, or non-steroidal anti-inflammatory agents were excluded. No patients had symptomatic coronary artery diseases or a family history of premature cardiovascular diseases.

The study was carried out according to the Declaration of Helsinki and approved by Institutional Ethic Review Boards of the University Hospital of Verona, Italy and University Hospital “Policlinico di Bari”, Bari, Italy.

### Peripheral Blood Mononuclear Cells (PBMC) isolation

Fifteen ml whole blood were collected from all subjects included in the study. For CKD-HD patients the biological material was drawn before the second dialysis of the week. PBMC were isolated by density separation over a Ficoll–Paque (GE healthcare, Sweden) gradient (460 g for 30 min) and washed three times with PBS pH 7.4/1 mM EDTA (Sigma, Milan, Italy). Cells were counted, and viability was assessed by trypan blue exclusion method (>90% PBMCs were viable).

### RNA extraction and Real-Time PCR

Total RNA was isolated from PBMC by RNeasy mini kit Qiagen (QIAGEN AG, Basel, Switzerland) and quantified by Quant-iT RNA Assay kit with Qubit Fluorometer (Invitrogen).

Reverse transcription of RNA was performed using the High Capacity cDNA Reverse Transcription Kit (Applied Biosystems), following the manufacturer’s instructions. One μg of RNA was reverse transcribed using random primer and MultiScribe Reverse Transcriptase. Real-time PCR amplification reactions were performed in duplicate in 20 μl of final volume via SYBR Green chemistry on ABI-Prism 7700 (Applied Biosystem). PCR protocol was performed using QuantiTect Primer Assays (Qiagen, Basel, Switzerland) for IL-1β, ASC, CASP-1, IL-18, NLRP3, CARD8, P2XR7 and β-actin: 50°C for 2 min, 95°C for 2 min and 40 two-step cycles: 95°C for 15 sec and 60°C for 30 sec.

Universal master mix obtained from Kapa Biosystems included all reagents. The β-actin gene amplification was used as a reference standard to normalize the target signal. The comparative Ct method (ΔΔCt) was used to quantify gene expression, and the relative quantification was calculated as 2^-ΔΔCt^. Amplification specificity was controlled by a melting curve analysis and the amount of mRNA target was evaluated using the comparative Ct method.

### Mitochondrial reactive oxygen species (ROS) measurement

Freshly isolated PBMC were incubated with 5 μM MitoSOX Red mitochondrial superoxide indicator (Molecular Probes, Invitrogen, Carlsbad, CA), for 10 min at 37°C, in the dark, according to manufacturer’s instructions. Autofluorescence of unstained cells were used as control for each sample. Approximately 40,000 gated events were acquired for each sample on a FACSCanto (Becton Dickinson, San Jose, CA) and analyzed using FlowJo software (TreeStar, Ashland, OR). Dead cells and debris were excluded based upon forward scatter and side scatter measurements. All analyses were gated on PBMC, based on morphologic identification (forward scatter vs. side scatter). Percentage of Mitosox-positive cells (fluorescence signals at 580 nm) was calculated by subtracting the percentage of autofluorescence-positive cells.

### Confocal microscopy

Freshly isolated PBMC from 3 NORM and 3 CKD-HD were spotted on poly-L-lysine-coated slides and incubated with Mitotracker deep red (Molecular Probes, Invitrogen, Carlsbad, CA), 100 nM for 30 min at 37°C according to manufacturer’s instruction. The cells were washed and fixed with 4% paraformaldehyde (PFA). PFA was quenched with 50 mM NH_4_Cl. Cells were then permeabilized with PBS-0.1% Triton X-100. After blocking with 1% bovine serum albumin in PBS, the slides were incubated with anti-NLRP3 and anti-ASC antibodies (Abcam) for 1 hour. The slides were then extensively washed in PBS and incubated with Alexa Fluor 488 Goat Anti-Rabbit and Alexa Fluor 594 anti-mouse. Finally, nuclei were stained with DAPI. Negative controls were performed by omitting the primary antibodies (S1 Fig.). Images were collected using the SP5 confocal microscope from Leica Microsystems (Wetzlar, Germany).

### Cell culture

Freshly isolated PBMC from 3 randomly selected CKD-HD were cultured in RPMI 1640 medium (Sigma) supplemented with 2 mM L-glutamine, penicillin (100 U/ml) and streptomycin (100 μg/ml) and stimulated with LPS (10 ng/ml) (Sigma) for 4 h followed by ATP (Sigma) 1 mM for 15 min. MitoTEMPO (100 μM) (Santa Cruz) was added to the medium 1 h before LPS priming.

### Western blot

Cells derived from 10 NORM and 10 CKD-HD were lysed in RIPA buffer (1 mM phenylmethylsulphonylfluoride, 5 mM EDTA, 1 mM sodium orthovanadate, 150 mM sodium chloride, 8 μg/ml leupeptin, 1.5% NonidetP-40, 20 mM Tris–HCl, pH 7.4). Aliquots containing 45 μg of proteins from each lysate were subjected to SDS–PAGE on a Criterion Tris-HCl 4–20% precast gels and then transferred onto PVDF membrane (Millipore). Membranes were incubated with primary antibodies as follows: anti-cleaved-Caspase-1 (Cell Signaling), anti-proCaspase-1 (Cell Signaling), anti-cleaved-IL1β (Cell Signaling), anti-pro-IL-1β (Santa Cruz), anti-IL-18 (Santa Cruz), and anti-actin (Santa Cruz). Blots were subsequently incubated with secondary antibodies HRP-labelled (Santa Cruz Biotechnology Santa Cruz, CA). Proteins were detected by Chemiluminescence (Amersham, GE Healthcare). Images were acquired using a scanner EPSON Perfection 2580 Photo (EPSON, Long Beach, CA, USA) and quantified by Image J 1.34 Software (http://rsb.info.nih.gov/ij/). The intensity of bands of interest was normalized to the signal intensity of the corresponding procaspase-1, pro-IL-1β, or pro-IL-18 band present on the same membrane. Actin was used as loading control.

### Microarray analysis

For all 22 patients (17 CKD-HD and 5 NORM) included in the microarray-group, RNA was processed and hybridized to GeneChip Human Genome U133A oligonucleotide microarray (Affymetrix, Santa Clara, CA) which contains 22,283 gene probe sets, representing 12,357 human genes, plus approximately 3,800 expressed sequence tag clones (ESTs), according to manufacturer’s instructions. We used the default settings of Affymetrix Microarray Suite software version 5 (MAS 5.0; Affymetrix) to calculate scaled gene expression values. Results of the microarray experiments are available in Gene Expression Omnibus (Accession Number GSE15072).

### Statistical analysis

Data are expressed as the mean ± standard deviation (SD). T-test and chi-square test were used to assess differences in clinical and demographic features. A value of p < 0.05 was considered to be statistically significant.

For microarray analysis, gene expression values for the 22,283 gene probe sets, scaled to the target intensity of 2,500, were log transformed. Probe sets unexpressed in the entire microarray cohort of patients were omitted. However, for our analysis we used only 14 gene probe sets (corresponding to 5 genes) encoding for the biological elements included in the NLRP3 inflammasome according to NOD-like receptor signaling pathway in KEGG pathway analysis (http://www.genome.jp/kegg/pathway.html). Principal component analysis (PCA) was performed using Spotfire decision site 9.0 (www.spotfire.com). A p value cut-off of 0.005 was used to identify differentially expressed probe sets between the two groups.

## Results

### Increased expression of genes encoding for NLRP3 inflammasome components and pro-inflammatory cytokines in PBMC from CKD-HD patients

**Fig 1:**
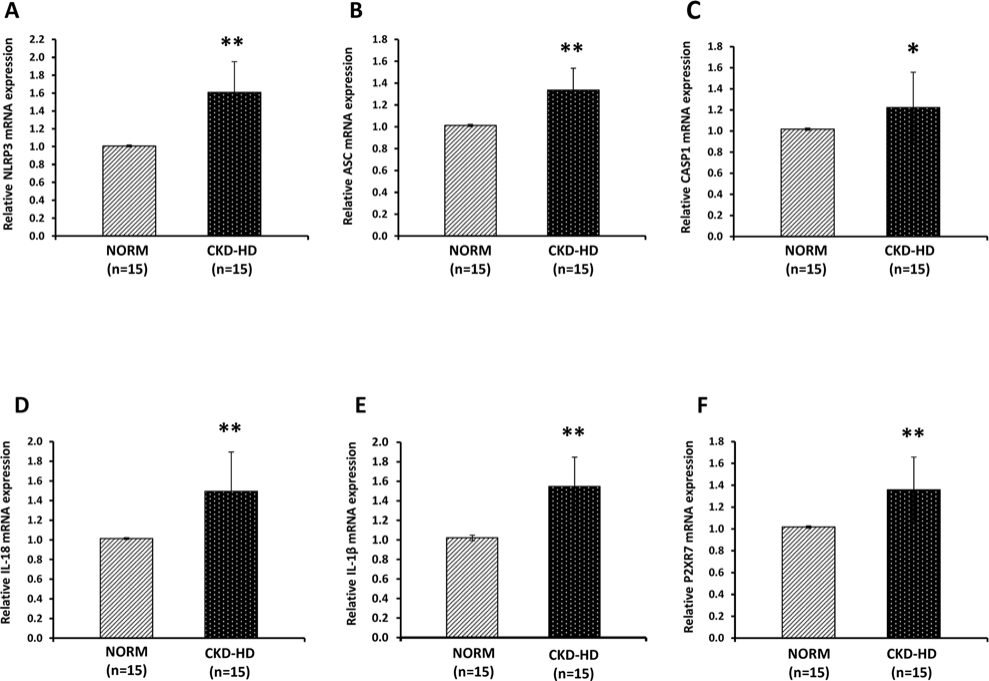
NOD-like receptor 3 (NLRP3), apoptosis-associated speck-like protein containing a CARD domain (ASC), caspase-1 (CASP-1), IL-1β, IL-18 and P2XR7 gene expression by Real-Time PCR in PBMC from healthy subjects (NORM) and hemodialyzed chronic kidney disease patients (CKD-HD). Histograms represent the mean ± SD of (A) NLRP3, (B) ASC, (C) CASP-1, (D) IL-18, (E) IL-1β and (F) P2XR7 mRNA levels determined by Real-Time PCR in PBMC isolated from 15 NORM and 15 CKD-HD patients. For all genes, expression levels resulted significantly higher in CKD-HD compared to NORM (*p<0.05, **p<0.01).

RT-PCR demonstrated that mRNA levels of *NLRP3*, *ASC*, *CASP-1* (inflammasome components) and pro-inflammatory cytokines IL-1β and IL-18 were higher in PBMC isolated from CKD-HD patients compared to NORM (Fig. 1).

P2X7 receptor (P2X7R), an ATP-gated cation channel that plays a key role in ATP-induced inflammasome activation [44] was up-regulated in patients.

*CARD 8*, an inhibitor of the aforementioned inflammasome, resulted down-regulated in CKD-HD versus controls (S2 Fig.).

These results reveal an enhanced inflammatory status in patients with severe renal failure undergoing dialysis treatment.

### Increment of mitochondrial ROS level in CKD-HD compared to controls

To measure the mitochondrial ROS production in PBMC of CKD-HD patients, we used the MitoSOX Red, a fluorogenic dye for selective detection of superoxide in mitochondria of live cells.

**Fig 2:**
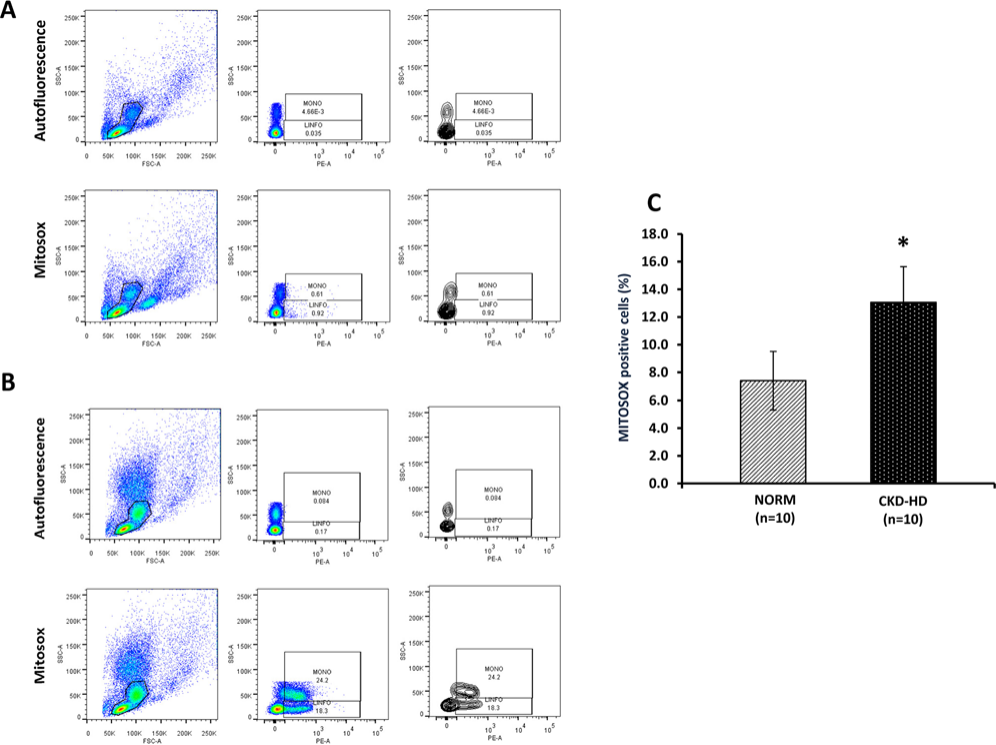
Mitochondrial ROS production by FACS in PBMC from healthy subjects (NORM) and hemodialyzed chronic kidney disease patients (CKD-HD). Freshly isolated PBMC from 10 NORM and 10 CKD were incubated with 5 μM Mitosox RED for 10 min at 37°. After centrifugation at 4°C the cells were analyzed for Mitosox signal in a flow cytometer. (A and B) Representative FACS image of PBMC from one NORM and one CKD-HD patient, respectively. (C) Histogram indicates median value ± SD of Mitosox positive cells in CKD-HD and NORM. CKD-HD patients showed higher number of positive cells compared to NORM (*p<0.05).

CKD-HD patients showed a significant higher levels of mitochondrial ROS compared to NORM (Fig. 2).

This result was in line with our previous findings demonstrating a CKD-related mitochondrial dysfunction [45] and it suggests a possible link between this organelle and inflammasome activation.

### Colocalization of NLRP3, ASC and mitochondria in PBMC from CKD-HD patients

**Fig 3:**
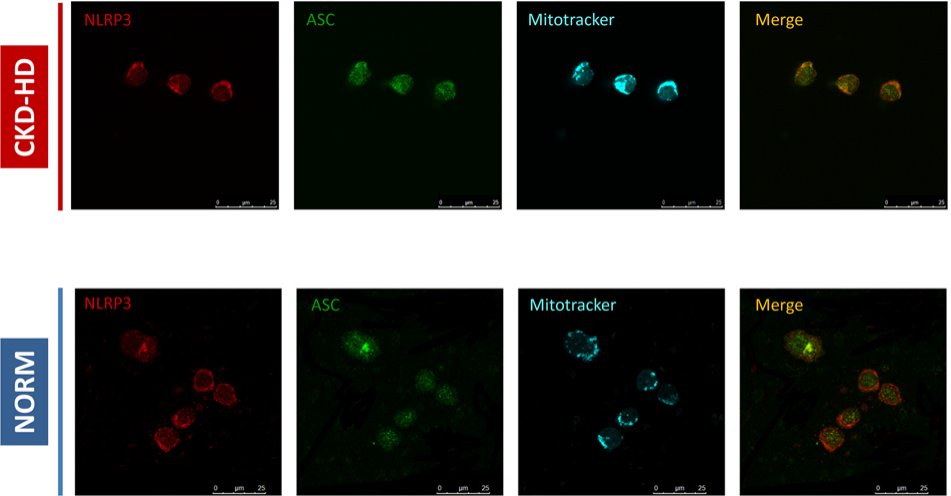
Colocalization of NLRP3, ASC and mitochondria in PBMC from hemodialyzed chronic kidney disease patients (CKD-HD). Freshly isolated PBMC from 3 NORM and 3 CKD-HD were spotted on poly-L-lysine-coated slides and incubated with Mitotracker deep red (100 nM) for 30 min at 37°C. The cells were washed and fixed with 4% PFA. After blocking with BSA 1%, the slides were incubated with anti-NLRP3 and anti-ASC antibodies. Then, the cells were incubated with secondary antibodies and nuclei were stained with DAPI. The figure shows representative confocal image for NLRP3, ASC and mitochondria in PBMC isolated from (*upper*) one CKD-HD patient and (*lower*) one NORM. NLRP3 protein co-localizes with ASC and mitochondria in PBMC from CKD-HD patients, whereas it remains in cytoplasmic granular structure in PBMC from NORM.

ROS are short-lived molecules and they can act as a signaling messenger only for a short distance [46]. Thus, to be effective NLRP3 should be localized in close proximity to mitochondria allowing an efficient sensing of the ROS produced by this organelle. To demonstrate the above mentioned co-localization and to evaluate the activation of NLRP3 inflammasome in CKD-HD patients, we performed confocal microscopy experiments using MitoTracker Deep Red FM, a far red-fluorescent dye that stains mitochondria in live cells. Interestingly, NLRP3 protein co-localizes with ASC and mitochondria in PBMC from CKD-HD patients whereas it remains in cytoplasmic granular structure in PBMC from NORM (Fig. 3).

These results show the activation of NLRP3 inflammasome in PBMC from CKD-HD patients with a possible involvement of mitochondria.

### Caspase-1 activation and pro-inflammatory cytokines maturation in PBMC from CKD-HD patients

Since the NLRP3 inflammasome once activated causes the proteolytic cleavage of caspase-1 and the subsequent maturation of pro-inflammatory cytokines [36], we decided to measure the protein level of activated (cleaved) caspase-1 and mature IL1β and IL-18 in 10 CKD-HD patients and 10 NORM from the training-group.

**Fig 4:**
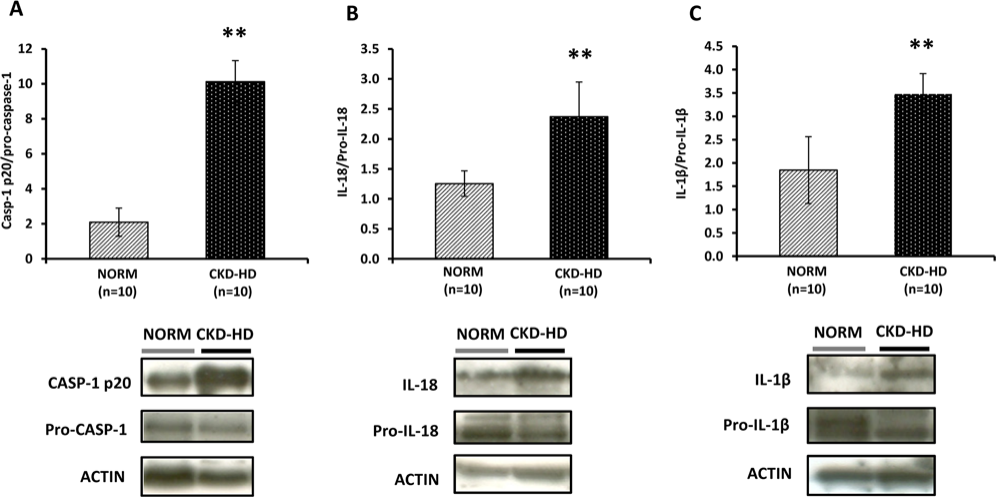
Mature (cleaved) forms of Caspase-1, IL-1β and IL-18 in PBMC from healthy subjects (NORM) and hemodialyzed chronic kidney disease patients (CKD-HD). Histograms represent the mean ± SD of (A) cleaved caspase-1, (B) IL-18 and (C) IL-1β protein levels, measured by western blotting, in total cell lysates of PBMC isolated from 10 NORM and 10 CKD-HD. In the bottom, representative western blotting experiments. Protein levels were significantly higher in CKD-HD patients compared to NORM (**p<0.01).

As predictable, the protein levels of caspase and both cytokines were higher in CKD-HD patients compared to NORM (p<0.01) (Fig. 4).

### Inhibitory effects of mitoTEMPO on caspase-1 activation and maturation of IL-1β and IL-18 induced by LPS/ATP in PBMC from CKD-HD

To better assess the role of mitochondria in inflammasome activation, we measured the level of CASP-1, IL-1β and IL-18 in PBMC from CKD-HD stimulated with LPS/ATP, Pathogen-associated molecular pattern (PAMP) and danger-associated molecular pattern (DAMP) signals able to activate NLRP3 inflammasome, in absence or presence of mitoTEMPO, an inhibitor of mitochondrial ROS production.

**Fig 5:**
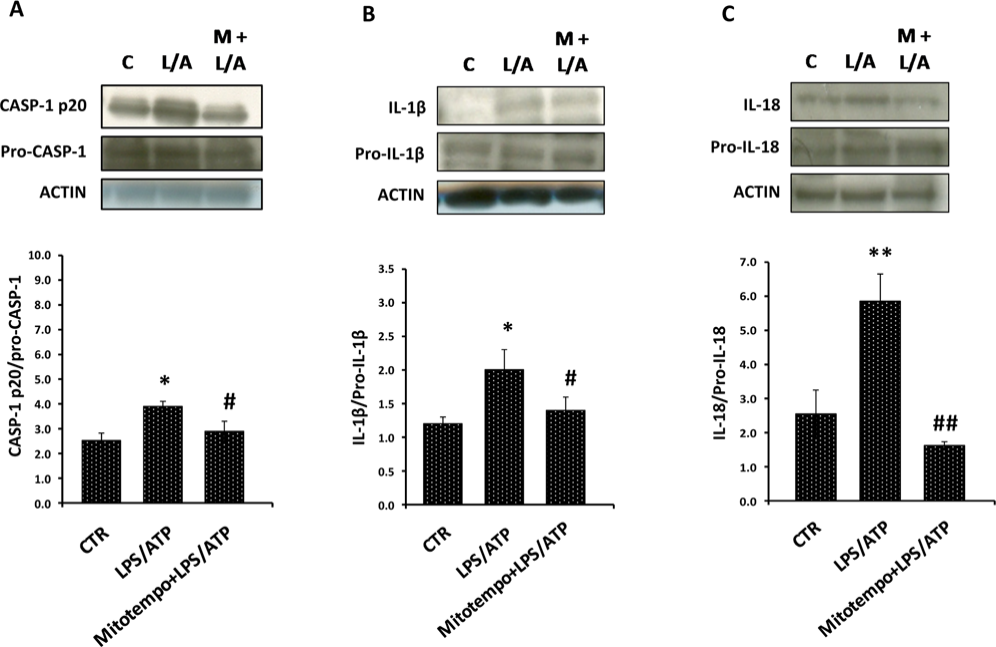
Inhibitory effects of mitoTEMPO on caspase-1 activation and maturation of IL-1β and IL-18 induced by LPS/ATP in PBMC from hemodialyzed chronic kidney disease patients (CKD-HD). PBMC from 3 CKD-HD were primed with LPS (10 ng/ml) for 4 h in presence or absence of MitoTEMPO (100 μM) and stimulated with ATP (1 mM) for 15 minutes. Histograms represent the mean ± SD of (A) cleaved caspase-1, (B) IL-1β and (C) IL-18 proteins level in total cell lysates. The intensity of each protein was quantified and normalized to the signal intensity of the corresponding procaspase-1, pro-IL-1β, or pro-IL-18 band present on the same membrane. In the upper part, representative western blotting experiments for (A) CASP-1, (B) IL-1β and (C) IL-18. In all experiments, MitoTEMPO caused a reduction on caspase-1 activation and maturation of IL-1β and IL-18 induced by LPS/ATP. *p<0.05, **p<0.01 vs CTR; #p<0.05, ##p<0.01 vs LPS/ATP.

As shown in Fig. 5, mitoTEMPO caused a reduction of caspase-1 activation and cytokines maturation in CKD-HD.

These results confirmed the close association between inflammation and mitochondrial dysregulation in CKD-KD patients.

### Microarray analysis confirmed a de-regulation of inflammasome genes in CKD-HD patients compared to healthy subjects

Microarray analysis revealed that 5 up to 15 gene probe sets were able to discriminate CKD-HD from NORM (cut-off of discrimination: p<0.005).

**Fig 6:**
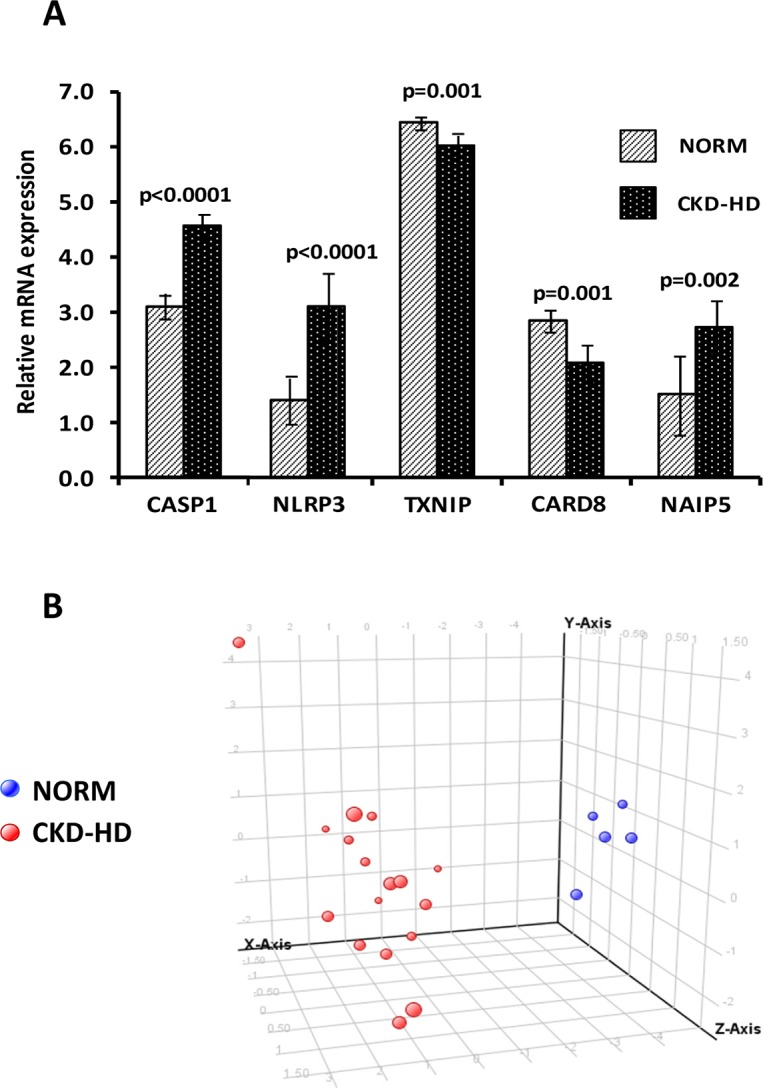
NLRP3 inflammasome genes differentially expressed in PBMC from healthy subjects (NORM) versus hemodialyzed chronic kidney disease patients (CKD-HD) and principal component analysis (PCA) discriminating patients from controls. (A) Histogram shows the normalized level of expression of *CASP1*, *NLRP3*, *NAIP5*, *TXNIP* and *CARD8* in CKD-HD and NORM. (B) PCA, built using the expression level of the above-mentioned genes, demonstrates the degree of discrimination in three dimensional space of the study groups. Blue dots indicate NORM and red dots CKD-HD patients.

Significantly up-regulated genes in CKD-HD were *CASP1*, *NLRP3* and *NAIP5*, while those down-regulated were *TXNIP* and *CARD8* (Fig. 6A).

Principal component analysis (PCA) using the expression levels of the aforementioned gene probe sets was able to clearly discriminate in three dimensional space CKD-HD patients from NORM (Fig. 6B).

## Discussion

In this study, performed by using classical bio-molecular methodologies (RT-PCR, western blot, FACS analysis, confocal microscopy) combined with an high-throughput technology (microarray), we found that the NLRP3 inflammasome was activated in immunocompetent peripheral cell lines isolated from uremic patients undergoing dialysis treatment.

This result was in line with a previous study showing the inflammasome activation in kidney tissue of mice with renal impairment followed to unilateral ureteral obstruction. Compared with wild-type, Nlrp3^-/-^ mice had less tubular injury, inflammation and fibrosis associated with a reduction in caspase-1 activation and maturation of IL-1β and IL-18 [47].

Additionally *in vitro* studies have shown that elevated levels of IL-1β and IL-18, produced during CKD, were able to promote renal tubulointerstitial fibrosis [48,49]. In particular, stimulation of primary cultures of human renal fibroblast with IL-1β for 24 h caused, collagen type 1 production and secretion of fibronectin and transforming growth factor-β (TGF-β).

Similarly, tubular proximal epithelial cells stimulated with IL-18 showed increase alpha-smooth muscle actin (α-SMA) expression, type I collagen and fibronectin production in dosage- and/or time-dependent manners.

Several papers, then, indicate that danger-associated molecular patterns (DAMPs) such as extracellular ATP, ROS, extracellular matrix components, are capable to activate NLRP3 inflammasome in several renal diseases [39,50–54].

More recently, Zhuang et al demonstrated a pathogenic role of NLRP3 inflammasome/caspase-1/mitochondria axis in mediating albumin-induced renal tubular injury [55]. Likewise, mitochondrial ROS and NLRP3 inflammasome activation aggravate diabetic nephropathy in non myeloid-derived cells [56].

Additionally, in the current study, we found elevated production of mitochondrial ROS in PBMC from CKD-HD patients confirming mitochondrial impairment we previously reported [45]. In fact, using an innovative high-throughput technology, we discovered that several biological elements involved in the oxidative phosphorylation system (OXPHOS) and two key constituents of the mitochondrial complex IV (COXI and COXIV) were deregulated in CKD and HD patients compared to the healthy controls.

Furthermore, complex IV activity, the terminal enzyme of the mitochondrial respiratory chain catalyzing the electron transfer from reduced cytochrome c to oxygen [57], resulted significantly lower in CKD-HD patients compared to healthy controls demonstrating a reduced activity of OXPHOS in this population.

It is conceivable that ROS produced by damaged organelles, in our patients’ population, could be one of the abovementioned signal 1 in NLRP3 inflammasome activation.

In fact, in this paper, colocalization of NLRP3/ASC/mitochondria together with the inhibitory effect of mitoTEMPO (an inhibitor of mitochondrial ROS production) on CASP-1 maturation and cytokines activation in PBMC from CKD-HD patients confirm the involvement of this organelle.

This effect has been previously demonstrated through two biological/biochemical mechanisms that cause generation of ROS: inhibition of complex I or III of the mitochondrial respiratory chain and inhibition of mitophagy/autophagy resulting in the prolonged presence of damaged mitochondria [58–60].

Contrarily, Schreiber et al [61] have recently found ROS to be potent suppressors of inflammasome. In particular, Phox-generated ROS downregulate caspase 1, thereby keeping the inflammasome in check and limiting antineutrophil cytoplasmic antibody (ANCA)-induced inflammation.

Notably, although extremely interesting, these unexpected data have been obtained, by a fashionable set of experiments, in a mouse model of ANCA-associated crescentic glomerulonephritis. However, because of the extreme complexity of the disease and the lack of etiopathogenetic information, no patients with this disease have been included in our study.

Moreover, microarray and principal component analysis, showed that 5 up to 15 gene probe sets were able to discriminate CKD-HD from NORM.

Significantly up-regulated genes in CKD-HD were *CASP1*, *NLRP3* and *NAIP5*, while those down-regulated were *TXNIP* and *CARD8* (Fig. 6A).

Interestingly, caspase recruitment domain–containing protein (CARD) 8 (also known as tumor CARD-containing antagonist of caspase nine-TUCAN), resulted down-regulated. This protein interacts physically with caspase-1 and negatively regulates caspase-1-dependent IL-1β expression [62,63]. A down-regulation of this protein has been reported in a model of human pancreatic islets isolated for transplantation exposed to oxidative stress. Authors suggested that an increased production of ROS and subsequent oxidative stress, through CARD8 down-regulation, represents a possible mechanism by which high concentrations of glucose could kill beta cells [64].

However, limitations of this part of the study is the unusual bioinformatic analysis (because the low number of patients we did not perform false discovery rate evaluation) and the unbalance between patients and healthy subjects.

All together these data showed, for the first time, that NLRP3 inflammasome was activated in uremic patients undergoing dialysis treatment and they suggested that this unphysiological condition could be possibly induced by mitochondrial dysfunction.

Finally, NLRP3 inflammasome pathway could turn to be a valuable therapeutic target to minimize or avoid severe clinical complications in chronic kidney disease patients with advanced renal impairment.

## Conclusions

In conclusion, the present study reveals, for the first time, that damaged mitochondria of uremic patients through an elevated production of ROS could be able to activate NLRP3 inflammasome representing a new deregulated biological machinery and a novel therapeutic target in CKD-HD patients.

## Supporting Information

S1 Fig Control for nonspecific staining (NLRP3 and ASC primary antibodies). (TIF)

S2 Fig Caspase recruitment domain–containing protein (CARD) 8 gene expression by Real-Time PCR in PBMC from healthy subjects (NORM) and hemodialyzed chronic kidney disease patients (CKD-HD). Histogram represents the mean ± SD of CARD8 mRNA level determined by Real-Time PCR in PBMC isolated from 15 NORM and 15 CKD-HD patients. Expression levels resulted significantly higher in CKD-HD compared to NORM.
(TIF)
