# Supplementary figures and images for: NLRP3 Inflammasome Activation in Dialyzed Chronic Kidney Disease Patients
Source: PLoS One. 2015 Mar 23;10(3):e0122272. doi: 10.1371/journal.pone.0122272 (PMC4370586; doi:10.1371/journal.pone.0122272)

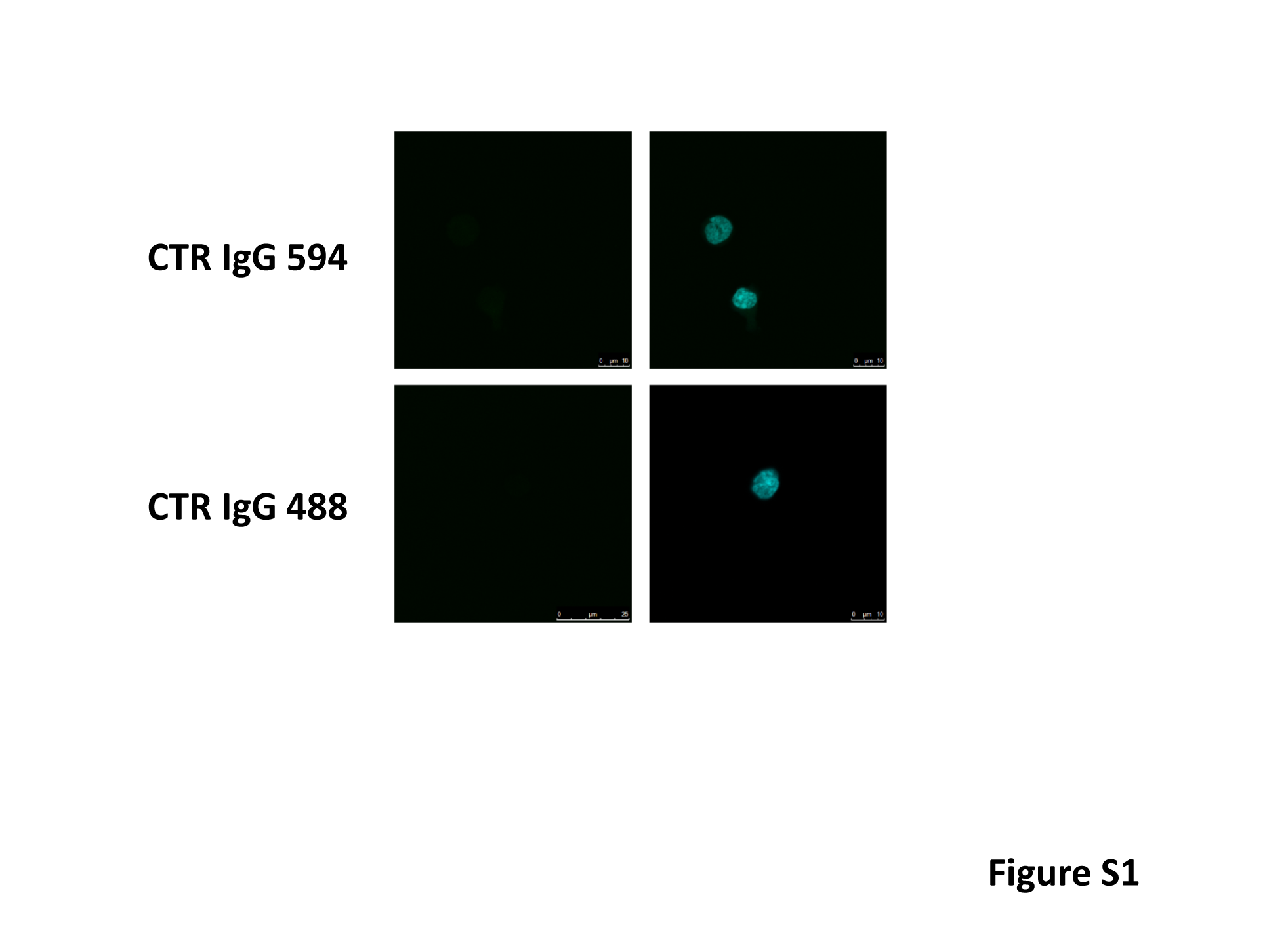

Supplement: S1 Fig — (TIF) [file pone.0122272.s001.tif]

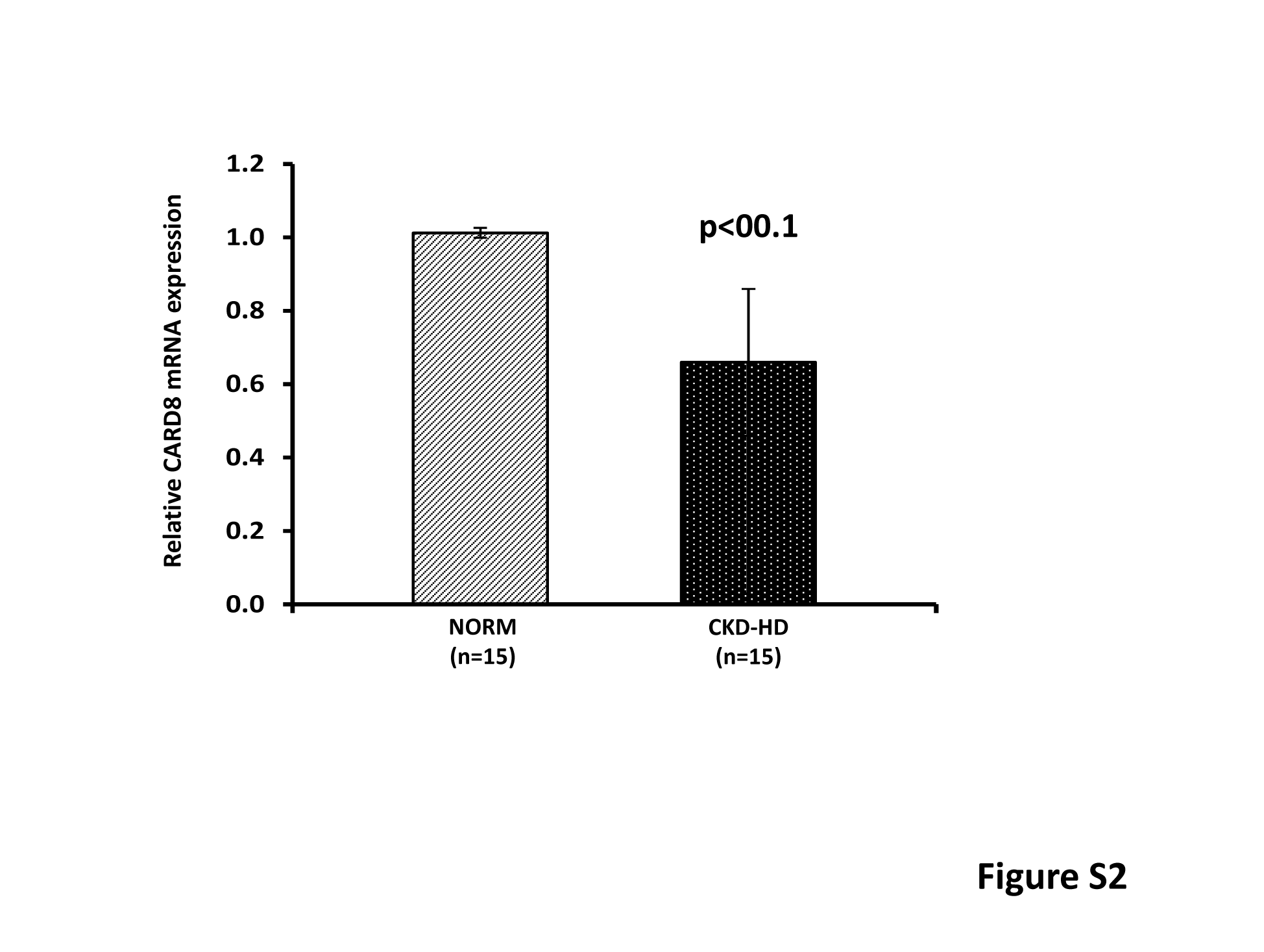

Supplement: S2 Fig — Histogram represents the mean ± SD of CARD8 mRNA level determined by Real-Time PCR in PBMC isolated from 15 NORM and 15 CKD-HD patients. Expression levels resulted significantly higher in CKD-HD compared to NORM. (TIF) [file pone.0122272.s002.tif]
